# The influence of maturity on recovery and perceived exertion, and its relationship with illnesses and non-contact injuries in young soccer players

**DOI:** 10.5114/biolsport.2022.109953

**Published:** 2021-10-25

**Authors:** Mauro Mandorino, Antonio J. Figueiredo, Giancarlo Condello, Antonio Tessitore

**Affiliations:** 1 Department of Movement, Human and Health Sciences, University of Rome “Foro Italico”, Rome, Italy; 2 University of Coimbra, Faculty of Sport Sciences and Physical Education, Research Unit for Sport and Physical Activity, Coimbra, Portugal; 3 Department of Medicine and Surgery, University of Parma, Italy

**Keywords:** Youth, Fatigue, Maturity, Workload, Recovery

## Abstract

A high training load could increase the risk of injury in soccer players. However, there is still a lack of knowledge regarding the effect on young athletes, in whom a different maturity status could lead to different physiological responses to training. Therefore, the aim of this study was to investigate the association of internal load markers and recovery status with risk of illnesses and non-contact injuries in young soccer players characterised by different maturity status. Twenty-three U14 soccer players were monitored during a full season and categorised according to years from peak height velocity (PHV). Based on the estimated values, athletes were divided into three different groups: Pre-PHV, Circa-PHV and Post-PHV players. The following internal load markers were monitored: rating of perceived exertion (RPE), session rating of perceived exertion (S-RPE), weekly load (WL), cumulative loads for 2, 3, and 4 weeks (WL2, WL3, WL4), acute to chronic workload ratio for 2, 3 and 4 weeks (A:C2, A:C3, A:C4) and week-to-week percentage variation (%WL). Recovery status was quantified using the total quality recovery (TQR) scale. Z-score transformation was adopted for TQR and RPE values and the difference between the parameters was calculated (Z-TQR-RPE). The Kruskal-Wallis test was adopted to evaluate differences in TQR and RPE with respect to maturity offset. A multinomial regression analysis was performed to evaluate the association between internal load markers and risk of illness and non-contact injuries. The variables that showed a significant association were included in the receiver operating characteristic (ROC) curve analysis. The results revealed that RPE was significantly higher (*p* < 0.01) and TQR significantly lower (*p* < 0.05) in Post-PHV compared to Pre- and Circa-PHV. Moreover, RPE, A:C4, TQR and Z-TQR-RPE showed a significant (*p* < 0.01) association with non-contact injuries. The internal load markers included in ROC curve analysis showed poor predictive ability (AUC ≤ 0.6). A rapid increase in training load together with a decrease in recovery status may produce higher susceptibility to illnesses and non-contact injuries. The contrasting physiological responses found in relation to maturity status could explain the different injury predisposition in young soccer players.

## INTRODUCTION

Although sport practice is globally recognised to be positively associated with physical health benefits in children and adolescents [1], soccer participation could increase the risk of injuries [2]. The occurrence of injuries could induce harmful secondary effects as time loss from sport participation [3], injury-related talent development stagnation [4] and dropout [5]. Thus, considering the negative effects related to injuries, the prompt introduction of prevention strategies is crucial in order to reduce the injury burden and to maximise the performance [6]. However, according to the “sequence of prevention” model [7], before developing a prevention programme, it is necessary to understand the related injury risk factors.

Injury is a complex multifactorial phenomenon determined by the interaction of extrinsic and intrinsic factors, which in turn can be divided into modifiable (e.g., strength, flexibility) and non-modifiable ones (e.g., sex, age, maturity status) [8]. However, according to the model introduced by Windt & Gabbett [9], the interaction of all these factors (modifiable and non-modifiable) is not sufficient to induce the onset of an injury. Indeed, according to the authors, the workload represents the main vehicle whereby an athlete moves from being a “predisposed athlete” to a “susceptible athlete” [9]. Therefore, as reported by Banister et al. [10], coaches and physical trainers should be aware that an imbalance between the negative function (fatigue model) and positive function (fitness model), produced by a training stimulus, could lead to side effects such injury, illness or overtraining. Accordingly, it is essential to identify which workload parameters could highlight an injury risk condition.

According to the UEFA Elite Club Injury Study teams [11], the internal load markers are recognised to be relevant in identifying risks of injury. Jaspers et al. [12] found an association between a higher cumulative workload and overuse injuries, while Fanchini et al. [13] observed an association between a rapid increase in workloads and risk of non-contact injuries. However, these studies are limited to adult players and cannot be generalised to youth soccer. Indeed, young athletes are generally exposed to different training volumes, and they also experience growing processes characterised by fast changes in hormonal release, height, weight and body composition [14]; all these factors could induce different responses to training. Therefore, young soccer players may exhibit a different susceptibility to injury with respect to adults, as evidenced by Pfirrmann et al. [15], who reported a higher training injury incidence in young male soccer players between 8 and 19 years of age (belonging to soccer academy, high level, and elite) compared to professional adult soccer players.

Puberty represents a critical time for young athletes due to the numerous physiological changes [16]. To date, several studies have conducted epidemiological analyses with the aim of investigating how these maturity processes could impact on injury risk, showing a higher injury rate both during and after the period of peak height velocity (PHV) [17–19]. The risk factors contributing to the rise in injury incidence are not completely clear. Different neuromuscular control [20], musculoskeletal characteristics such as joint stiffness [21], and physical performances [22] could explain the different predisposition to injury. However, to the best of our knowledge, to date no studies have investigated the training load in relation to the maturity status and its association with the risk of injuries and illnesses in young soccer players. Brink et al. [23] analysed the impact of physical stress and recovery on the risk of injury and illness in youth soccer, but without examining the maturity status of the players.

Therefore, considering the risk of injury associated with high training loads and the possible different physiological responses to training determined by different maturity status of the young players, the current study presents multiple aims: (1) to analyse the injury incidence according to players’ maturity status; (2) to analyse training responses in relation to maturity status; (3) to identify the association of internal load markers and recovery with the risk of injuries and illnesses. It was hypothesised that young soccer players with different maturity status could exhibit a different response to training, and consequently a different predisposition to injury.

## MATERIALS AND METHODS

### Participants

A prospective longitudinal cohort study design was employed to monitor internal load parameters, state of recovery, and history of injuries and illnesses in U14 soccer players. In this scope, 23 young soccer players (age = 13.5 ± 0.3 years, body mass = 51.5 ± 8.5 kg, height = 164 ± 7.3 cm) were monitored over one entire soccer season. Participants belonged to an U14 team, trained 3 days per week and competed once a week in an U14 sub-elite championship. Data were obtained from the club as players’ data were routinely collected over the course of the soccer season [24]. Before the commencement players’ parents/guardians signed their written informed consent to the data collection, which was conducted in accordance with the Declaration of Helsinki (2013) and approved by the local research ethics committee of the University of Rome “Foro Italico” (number CARD/64/2020).

### Variables collected

Injuries, illnesses, maturity offset, training load parameters, and recovery status of the players were collected during the season. The definitions of these variables and how they were calculated are summarised in Table 1.

**TABLE 1:** Summary of the variables collected during the season.

Variables	Definition	Calculation	Supporting literature
Injuries (contact and non-contact)	Contact and non-contact injuries were classified whether they occurred with or without physical contact between players	$\frac{\text{number of injuries}}{\text{training exposure}}\times 1000\text{ h}$	Fuller et al. [25]
			Yu & Garret [26]

Illnesses	Condition characterised by the presence of cold-related symptoms (e.g., influenza, fever, sore throat)	$\frac{\text{number of illnesses}}{\text{training exposure}}\times 1000\text{ h}$	Putlur et al. [27]

Maturity offset	The time before or after peak height velocity (PHV)	Maturity Offset = – 29.769 + 0.0003007 Leg Length and Sitting Height interaction – 0.01177 X Age and Leg Length interaction + 0.01639 Age and Sitting Height interaction + 0.445 Leg by Height ratio	Mirwald et al.[28]

S-RPE	Subjective internal training load	Training session rating of perceived exertion (RPE) training duration	Foster et al. [29]
			Impellizeri et al. [30]

WL, WL2, WL3, WL4	Cumulative training loads	Sum of the loads of all training sessions and matches over a period of one week (WL), two weeks (WL2), three weeks (WL3), four weeks (WL4)	Jaspers et al. [12]

A:C2, A:C3, A:C4	Acute to chronic workload ratio	$\frac{\text{acute workload (WL)}}{\text{chronic workload}}$	Gabbett et al. [43]
		Chronic loads were quantified as the rolling averages for 2, 3 and 4 weeks.	Fanchini et al. [13]

%WL	Week-to-week workload change	Percentage variation of the current WL respect to the previous WL	Fanchini et al. [13]

Z-TQR	Z-score transformation of the TQR values	$\frac{\text{individual players score-individual player average}}{\text{individual players standard deviation}}$	Gallo et al. [33]

Z-RPE	Z-score transformation of the RPE values	$\frac{\text{individual players score-individual player average}}{\text{individual players standard deviation}}$	Gallo et al. [33]

### Injuries and illnesses

Injuries were collected during the entire soccer season by the team’s physical therapist following the recommendations of the FIFA Consensus Model for Injury Registration [25]. An injury was reported if a player was unable to take full part in any future soccer activity participation [25] and its severity was defined as the number of days that a player was unable to fully participate in team training or match play. Therefore, injuries were classified as follows: slight (0 day); minimal (1–3 days); mild (4–7 days), moderate (8–28 days), severe (> 28 days). Injuries were also analysed in relation to the anatomic location, type, severity and mechanism (contact or non-contact injuries). Contact and non-contact injuries were classified according to whether they occurred with or without physical contact between players [26], while illnesses were collected by the medical staff and identified as a condition characterised by the presence of cold-related symptoms (e.g. influenza, fever, sore throat) [27].

### Maturity offset

The Mirwald et al. [28] algorithm was utilised to predict years from PHV, defined as maturity offset – MO (R = 0.94, R2 = 0.89, and SE = 0.59). A male-specific equation was employed. According to the maturity offset estimation, participants were classified in Pre-PHV (MO ≤ -0.3 years), Circa-PHV (MO between -0.3 and +0.3 years) and Post-PHV (MO ≥ +0.3 years) groups [19]. The mean chronological age, height, weight and MO values of the three groups are presented in Table 2.

**TABLE 2:** The mean chronological age, height, weight and MO values of Pre-, Circa-, and Post-PHV groups.

	Number of players	Chronological Age	Height (cm)	Body Mass (kg)	MO
**Pre-PHV**	9	13.3 ± 0.2	157.8 ± 5.8	43.5 ± 4.6	–0.9 ± 0.3
**Circa-PHV**	8	13.4 ± 0.3	167.4 ± 5.3	54.6 ± 4.4	0.0 ± 0.2
**Post-PHV**	6	13.6 ± 0.2	170.3 ± 2.9	59.4 ± 7.0	0.6 ± 0.2
**Total**	23	13.5 ± 0.3	164 ± 7.3	51.5 ± 8.5	–0.2 ± 0.7

MO = maturity offset; Pre-PHV = players with MO ≤ –0.3 years; Circa-PHV = players with MO between –0.3 and +0.3 years; Post-PHV = players with MO ≥ +0.3 years.

### Quantification of internal load and state of recovery

Training and match loads were quantified by means of the session-RPE method [29], validated in soccer by Impellizzeri et al. [30]. Accordingly, the players’ training and match session-RPE (S-RPE) scores were obtained by multiplying the duration of each training or match by the individual rate of perceived exertion (RPE) value assessed using the Borg CR-10 scale modified by Foster et al. [29]. The weekly load (WL) was calculated as the sum of the loads of all training sessions and matches over a period of one week. Cumulative loads were calculated for 2, 3, and 4 weeks (WL2, WL3, WL4). Monotony was quantified by dividing the mean daily load by the SD of the load over one week. Strain was calculated by multiplying monotony by the weekly load [29]. Chronic loads were quantified as the rolling averages for 2, 3 and 4 weeks. The acute (WL) to chronic (rolling average of previous weeks) workload ratio was separately calculated for 2, 3 and 4 weeks (A:C2, A:C3, A:C4) as previously reported by Fanchini et al. [13]. The relative week-to-week change was calculated as the percentage variation of the current WL with respect to the previous WL (%WL).

The players’ perceived recovery status was assessed before each training session and match, through the use of a modified 10-point total quality recovery (TQR) scale [31]. Athletes were asked to quantify their recovery status considering the psychophysical cues (i.e. mood states and muscle soreness) as suggested by the authors of the scale [32].

**FIG. 1:**
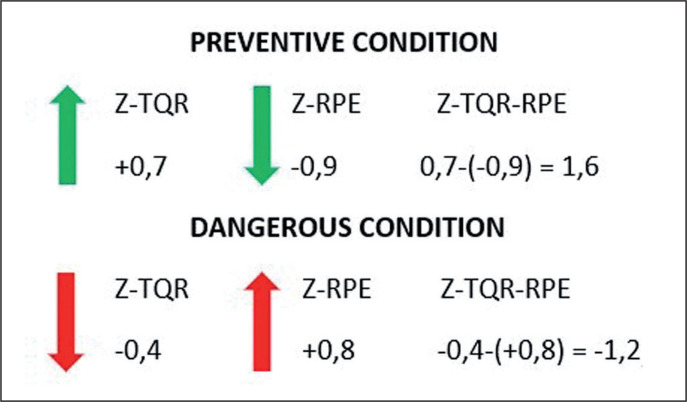
Z-TQR-RPE interpretation Z-score transformation was employed to evaluate daily variation of TQR and RPE respect to individual players average. Z-TQR and Z-RPE were combined through mathematical subtraction in order to quantify the variation of the state of recovery in relation to the variation of the perception of effort. Therefore, according to Kenttä & Hassmén [32] model, an increase in the training stress (RPE) associated with a lowering of the recovery status (TQR) was interpreted as a dangerous condition. Differently, a reduction in the daily perception of effort together with an increase in the recovery status was read as a preventive condition.

Overall, the RPE and TQR scores were reported in relation to the individual’s absolute average and normal variation recorded during the entire season. Thus, individual Z-scores (Z-TQR, Z-RPE) were calculated as follows: (individual players score – individual players average)/individual players standard deviation [33]. Z-score transformation was used to evaluate whether players’ daily TQR or RPE values were above or below the mean of the distribution. At this stage, the difference between Z-TQR and Z-RPE was calculated (Z-TQR-RPE). A negative result was interpreted as a dangerous condition characterised by a reduction in the recovery state and concomitant increase in the perception of effort compared to individual responses. A positive outcome was interpreted as a preventive factor with an increase in the state of recovery and reduction in the perception of effort. The model is further explained in Figure 1.

### Statistical analysis

Data were analysed using SPSS Statistics, version 25.0 (IBM Corp., Armonk, NY, USA). The level of statistical significance was set at p < 0.05 for all computations. Data are presented as mean ± standard deviations (mean ± SD).

Training and match injury incidence were calculated as the number of injuries per 1000 h of exposure in matches and training. The illness incidence was estimated as the number of illnesses per 1000 playing hours [23]. Injury burden was calculated as follows: number of injuries × average number of time-loss days per injury per 1000 hours of soccer exposure [34].

Difference between injury rates and injury burden was calculated along with 95% confidence intervals (95% CI) using Poisson distribution and test-based methods (MedCalc Software, Ostend, Belgium). Differences in RPE and TQR between Pre-, Circa and Post-PHV players were examined with the Kruskal Wallis test after the Shapiro-Wilk test revealed the non-normal distribution of the data. To avoid the duration of the training impacting on the perception of effort and state of recovery, only training sessions lasting more than 70 minutes were included for this type of analysis. Particularly, the recovery status was evaluated 24 or 48 hours after the training session. When statistically significant differences existed, Dunn–Bonferroni adjusted post hoc comparisons were adopted. To measure effect size for these differences, eta-squared was used. The following values to interpret effect sizes from eta-squared (η^2^) were employed: 0.01 < 0.06 (small effect), 0.06 < 0.14 (moderate effect) and ≥ 0.14 (large effect) [35].

A multinomial regression analysis was adopted to identify differences between healthy, illness, and non-contact injured groups for all internal load and state of recovery variables. The healthy group was set as the reference category. The odds ratios (ORs) and 95% CIs were estimated for the independent variables.

A preliminary variance inflation factors (VIF) analysis was performed to detect multicollinearity between independent variables; markers with VIF ≥ 10 were excluded from the analysis.

All the independent variables that showed a significant association with illness or non-contact injury risk following the multinomial regression model were included in the receiver operating characteristic (ROC) curve analysis and the area under the curve (AUC) was calculated to test their predictive ability. An AUC of 0.5 suggests no discrimination, between 0.51 and 0.69 is considered poor discrimination, 0.70–0.79 is acceptable, 0.8 to 0.9 is considered excellent, and more than 0.9 outstanding [36]. The selected markers were analysed in the ROC curve individually and in combination after estimation of predicted probabilities.

## RESULTS

### Injuries and illnesses

**FIG. 2:**
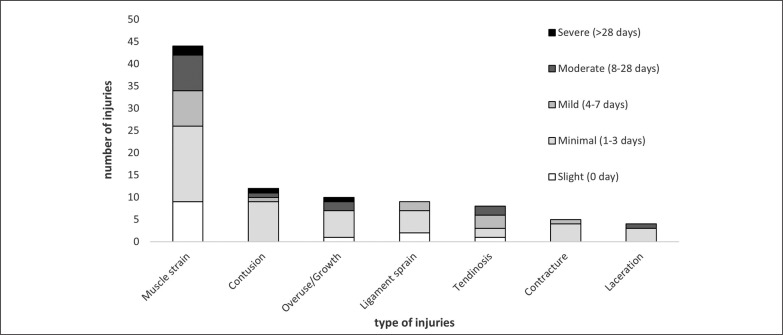
Injury type and severity of injuries Severity of injuries; Slight (0 day), Minimal (1-3 days), Mild (4-7 days), Moderate (8-28 days), Severe (>28 days).

**FIG. 3:**
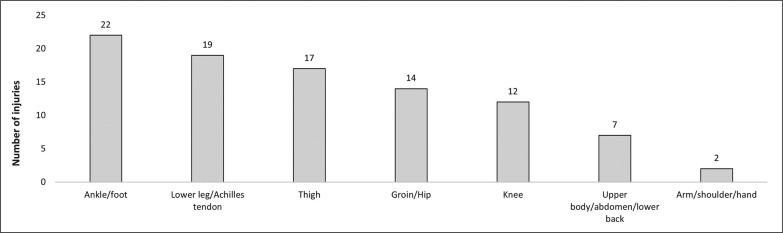
Anatomic location of injuries.

A total number of 93 injuries and 47 illnesses were recorded during the season. Of the 93 injuries, 17 (18.3%) were classified as contact injuries, and the remaining 76 (81.7%) as non-contact injuries. Additional information about severity, anatomic location and type of injuries is presented in Figures 2 and 3.

An overall injury incidence of 23.6 per 1000 playing hours was found. The injury incidence was significantly higher during matches than during training time (47.1 injuries per 1000 match hours [95% CI 30.2–70.1] vs 20.1 injuries per 1000 training hours [95% CI 15.7–25.5]; IRR = 2.3; p < 0.001). Moreover, when the injury incidence was calculated in relation to maturity status Circa-PHV players showed a significantly higher injury rate (31.2; 95% CI 22.5–42.2) compared to Pre-PHV players (18.3; [95% CI 12.1–26.6]; IRR = 1.7; p < 0.05). Although not statistically different, Post-PHV presented a higher injury rate (21.5; [95% CI 13.8–32.1]) compared to the Pre-PHV group.

Injury burden was also compared among the three maturity groups. Furthermore, a 90-day injury was identified as an outlier and excluded from the calculation in the Pre-PHV group.

Injury burden comparison revealed a significant difference between Circa-PHV and Pre-PHV players (128.7 injury days per 1000 h [95% CI 110.2–149.4] vs 82 injury days per 1000 h [95% CI 68–98]; IRR = 1.6; p < 0.01), between Post-PHV and Pre-PHV (149 injury days per 1000 h [95% CI 127.2–173.5] vs Pre-PHV; IRR = 1.8; p < 0.01) but not between Circa-PHV and Post-PHV players (Table 3).

**TABLE 3:** Injury incidence and injury burden respect to maturity offset

	Pre-PHV			Circa-PHV			Post-PHV		
	IR	CI (95%)		IR	CI (95%)		IR	CI (95%)	
**Injury incidence (1000h)**	18.3	12.1	26.6	31.2^a^	22.5	42.2	21.5	13.8	32.1
**Injury burden (1000h)**	82.0	68.0	98.0	128.7^b^	110.2	149.4	149.0^c^	127.2	173.5

Injury incidence and injury burden were calculated in relation to maturity offset (Pre-, Circa-, and Post-PHV).

a Statistical difference (p < 0.05) Circa-PHV vs Pre-PHV;

b Statistical difference (p < 0.01) Circa-PHV vs Pre-PHV;

c Statistical difference (p < 0.01) Post-PHV vs Pre-PHV; IR = injury rate; CI = confidence interval.

Regarding the illnesses, an overall incidence of 11.9 per 1000 playing hours was found. An incidence of 12.9, 14.9 and 7.2 was calculated in Pre-, Circa- and Post-PHV players, respectively. However, no differences were identified according to the maturity status.

### RPE and TQR analysis in relation to maturity offset

Data are presented as mean ± standard deviations (mean ± SD). RPE was significantly higher in Post-PHV compared with Pre-PHV (5.5 ± 1.8 arbitrary units (AU) vs 4.5 ± 1.7 AU, p < 0.01) and Circa-PHV (5.5 ± 1.8 AU vs 3.9 ± 1.6 AU, p < 0.01), after Dunn-Bonferroni post-hoc analysis. Moreover, Pre-PHV players showed higher RPE in comparison with Circa-PHV players (p < 0.01). A moderate effect was estimated (η^2^ = 0.13).

Instead, TQR was significantly lower in Post-PHV compared with Circa-PHV (6.6 ± 1.5 vs 6.9 ± 1.5, p < 0.05) and Pre-PHV (6.6 ± 1.5 vs 7.2 ± 1.4, p < 0.01) and in Circa-PHV compared with Pre-PHV (p < 0.01), after Dunn-Bonferroni post-hoc analysis. A small effect was estimated (η^2^ = 0.03).

### Multinomial regression analysis

Table 4 presents descriptive statistics for internal load and state of recovery parameters.

**TABLE 4:** Internal load markers in young soccer players

	Healthy	Illness	Non-contact injury
**RPE**	4.6 ± 1.8	4.2 ± 1.6	4.7 ± 1.9
**MONOTONY**	3.2 ± 3.4	3.2 ± 2.9	2.9 ± 2.7
**STRAIN**	5065.2 ± 5505.2	4629.8 ± 5531.2	4428.1 ± 3495.0
**S-RPE**	415.6 ± 250.2	358.1 ± 191.7	414.8 ± 222.3
**WL**	1633.2 ± 893.1	1410.0 ± 533.0	1564.9 ± 819.8
**A:C4**	1.1 ± 0.4	1.1 ± 0.3	1.2 ± 0.4
**TQR**	7.0 ± 1.4	6.9 ± 1.3	6.6 ± 1.4
**%WL**	13.8 ± 51.5	6.2 ± 21.3	11.7 ± 25.5
**Z-TQR-RPE**	0.02 ± 1.3	0.1 ± 1.2	–0.1 ± 1.4

Data are presented as mean ± SD. Scores of RPE, Monotony, Strain, S-RPE, WL, A:C4, TQR, %WL, Z-TQR-RPE in the week preceding an Illness, a non-contact injury and without injuries, were reported. RPE = rate of perceived exertion; S-RPE = session rating of perceived exertion; WL = weekly load; A:C4 = acute to chronic workload ratio for 4 weeks; TQR = total quality recovery; %WL = week-to-week percentage variation; Z-TQR-RPE = difference between Z-TQR (z-score transformation) and Z-RPE (z-score transformation).

Only illnesses and non-contact injuries were included in the model. The weeks preceding illnesses and non-contact injuries were compared to weeks without injuries [23]. Contact injuries, being by their nature unpredictable, were excluded from the following analysis [37].

WL2, WL3 and WL4, as well as A:C2, A:C3, showed multicol-linearity and therefore they were excluded from the regression analysis.

Multinomial regression analysis revealed an association between A:C4 (OR = 2.28 [95% CI 1.24–3.80]; p < 0.01), TQR (OR = 0.83 [95% CI 0.71–0.97]; p < 0.05), %WL (OR = 0.99 [95% CI 0.98–0.99]; p < 0.01] and risk of illnesses.

Moreover, RPE (OR = 1.27 [95% CI 1.13–1.44]; p < 0.01), A:C4 (OR = 4.39 [95% CI 2.84–6.79]; p < 0.01), TQR (OR =0.66 [95% CI 0.58–0.75]; p < 0.01) and Z-TQR-RPE (OR = 1.38 [95% CI 1.14–1.67]; p < 0.01) were associated with non-contact injuries.

### ROC curve analysis

The independent variables that showed an association with illnesses and non-contact injury risk were included in ROC curve analysis. A:C4, TQR and %WL displayed inability to predict risk of illness (AUC < 0.50). When the parameters were combined together, the AUC value rose to 0.58.

**FIG. 4:**
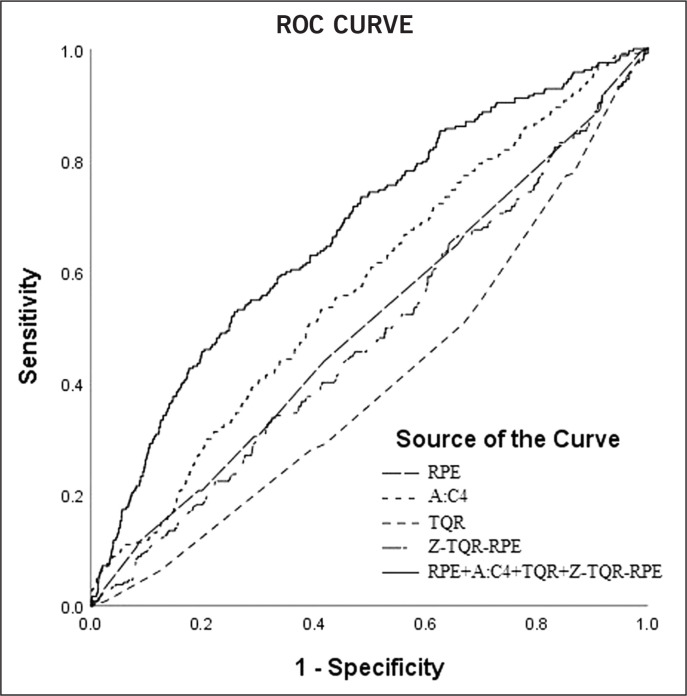
Receiving operating characteristic (ROC) curves for non-contact injuries ROC curves were presented for the single parameters (RPE, A:C4, TQR, Z-TQR-RPE) and in combination after predicted probabilities estimation (RPE+A:C4+TQR+Z-TQR-RPE).

Instead, RPE, TQR, A:C4 and Z-TQR-RPE presented AUC values of 0.51, 0.41, 0.57 and 0.48 respectively, regarding the predictive ability of risk of non-contact injuries. When combined, the AUC value rose to 0.63 (Figure 4).

## DISCUSSION

The aim of this study was to investigate how internal load parameters and recovery status impacted on risk of illness and injuries in young soccer players characterised by different maturity developmental levels. Particularly, Post- and Circa-PHV players presented a higher injury rate and injury burden compared to Pre-PHV players. Moreover, according to maturity offset, a different physiological response to training was found. Post- and Circa-PHV players exhibited a worse recovery ability, and Post-PHV players showed higher values of RPE.

Adopting the multinomial regression analysis, A:C4, TQR, %WL were identified as illness risk factors, while RPE, A:C4, TQR and Z-TQR-RPE were identified as non-contact injury risk factors. Starting from these results, it is possible to observe that risk of injury changed in relation to variation of internal load markers and recovery status. Therefore, the different physiological responses to training, identified according to maturity offset, might explain why a higher injury rate and injury burden were found in Circa- and Post-PHV compared to Pre-PHV players.

### Epidemiological analysis

The overall (contact and non-contact) injury incidence (23.6), the match injury incidence (47.1) and the training injury incidence (20.1) found in this study were comparable to previous ones [23, 38], but our training injury incidence was slightly higher. However, more than half of the injuries (63.4%) were classified as slight/minimal (0–3 days of absence).

When the injury incidence was investigated in relation to the maturity development, players classified as Circa-PHV had a higher incidence, in agreement with previous studies [17–19]. This moment during puberty known as the period of “adolescent awkwardness” [22] is characterised by a decline in performance and motor control. The impairment in coordination together with rapid growth in ligament, bone, and tendon structures (with different growth rates at a given moment) may subject young players to a higher risk of injuries.

The injury burden was also calculated in relation to the maturity status. Injury burden, defined as the cross-product of severity and incidence [34], is a very useful parameter that quantifies the impact of an injury on players [39]. In this regard, we found a significantly higher injury burden in Circa- and Post-PHV with respect to Pre-PHV, in line with Bult et al. [18]. This finding suggests that more mature players are subject to a higher number of injuries, which in turn are characterised by a greater severity. This may be understood by highlighting the increase of competition level and intensity in the age groups more auxologically compatible with a higher maturity status.

### Perception of effort and recovery status in relation to maturity offset

Analysing the perception of effort and recovery status in relation to the maturity offset, we found a greater perception in Post-PHV players compared to Pre- and Circa-PHV players. In addition, the Post-PHV players showed a worse recovery status, although only a small effect was identified. Puberty is a critical time for physical (e.g. body shape, body composition, height), hormonal and physiological changes [14]. Indeed, it has been well corroborated that the contribution of the different energy metabolisms changes in relation to the maturity status. In this regard, Ratel et al. [40] claimed that more mature athletes are able to maximise the use of the glycolytic system, differently from younger ones, who present reduced activity of the anaerobic metabolism due to a lower presence of phosphofructokinase. Indeed, less mature players present a lower concentration of glycolytic enzymes, lower muscle mass and lower ability to recruit type II muscle fibres. However, they exhibit higher oxidative phosphorylation enzyme activity and better ability to recruit type I muscle fibres [40]. In agreement with the study of De Morree et al. [41], we can speculate that the increased central command activity in the recruitment of muscle fibres, and the increase of the metabolic by-products (e.g. H+, Pi), may explain the higher perception of effort in post-PHV than in pre- and Circa-PHV players. In the same way, the faster rate of phosphocreatine resynthesises together with higher oxidative enzyme activity and greater proportion of type I fibres [40] may justify the best recovery ability of the Pre-PHV players as evidenced by higher TQR values before training sessions and matches.

Considering that more mature players express more power, speed and strength [42], they could be characterised by higher perception of effort and slower recovery ability, which could explain the higher injury rate and higher injury burden in Circa- and Post-PHV players compared to Pre-PHV players. However, in line with this evidence, it is not clear why Pre-PHV players exhibited higher perception of effort compared to Circa-PHV players. In this regard, we could speculate that athletes with a smaller and lighter “size” must increase energy expenditure to face more mature players who are stronger and heavier.

### Risk of illness and non-contact injury

One of the main goals for coaches and physical trainers is to identify all factors associated with injury risk. In our research we identified an association between internal load parameters (i.e., RPE, A:C4, %WL) and risk of illnesses and non-contact injuries, while monotony, strain and WL did not show any significant association (Table 5). In line with previous studies a high training load is associated with risk of illnesses [23]. Indeed, while moderate exercise could lead to benefits for the immune system [27], strenuous exercise could suppress several immune parameters [32].

**TABLE 5:** Multinomial regression analysis

	Illness			Non-contact injury		
	OR	CI (95%)		OR	CI (95%)	
**RPE**	1.04	0.89	1.20	1 27**	1.13	1.44
**S-RPE**	1.00	0.99	1.00	1.00	0.99	1.00
**MONOTONY**	0.93	0.82	1.05	1.11	0.97	1.28
**STRAIN**	1.00	1.00	1.00	1.00	1.00	1.00
**WL**	0.99	0.99	1.00	0.99	0.99	1.00
**A:C4**	2.28**	1.24	3.80	4.39**	2.84	6.79
**%WL**	0.99**	0.98	0.99	0.99	0.99	1.00
**TQR**	0.83*	0.71	0.97	0.66**	0.58	0.75
**Z-TQR-RPE**	1.09	0.88	1.35	1.38**	1.14	1.67

The internal load markers that presented a significant association in the multinomial regression analysis were reported. The healthy group was set as the reference category.

* Statistical difference (p < 0.05);

** Statistical difference (p < 0.01); OR = odds ratio; CI = confidence interval.

Moreover, our results showed an association between training load and risk of non-contact injuries in young soccer players. However, the problem is not the high training load per se, but rather its rapid increase (A:C4) [43]. A ‘spike’ in acute load relative to chronic load may subject athletes to an effort that they are not able to face, leading them to a state of fatigue [43]. Nevertheless, the usefulness of this parameter was recently questioned [44]. We must emphasize that in our study an association with non-contact injuries was identified, without any predictive ability. Future studies should clarify the use of this parameter as a training load monitoring tool.

In addition to the increase in RPE and A:C4, also a lowering in the recovery status, assessed through the TQR scale, was considered dangerous. The association between recovery status and risk of non-contact injuries has already been investigated in previous studies [23, 45] which used the REST-Q questionnaire. To the best of our knowledge, this is the first study to evaluate the association between injury risk and recovery status using the TQR scale. This scale may lead to several benefits, since it is quick and simple to submit, and it can be used daily, unlike the REST-Q questionnaire.

The novelty of this study is also represented by the introduction of a new parameter named Z-TQR-RPE. Z-score transformation was employed to analyse the daily variation of TQR and RPE compared to individual players’ scores. This parameter can provide variation within the recovery status in relation to the variation in perception of effort (Z-TQR-RPE). A positive value of Z-TQR-RPE was considered an optimal condition, determined by an increase in recovery status associated with a lowering in the individual perception of effort. Conversely, a negative value was interpreted as a “red flag” due to a decrease in recovery status accompanied by an excessive increase in the perception of effort. Indeed, it is possible to observe in Table 4 how Z-TQR-RPE decreases for the non-contact injury condition. However, future studies should verify whether this metric consistently maintains an association with the risk of injuries.

In summary, the findings of this study showed a significant association between internal load parameters, recovery status and risk of illnesses and non-contact injuries. However, according to Fanchini et al. [13], we need to underline that the concept of association is different from that of prediction. Indeed, the AUC values calculated indicate a poor predictive ability. Nevertheless, when the independent variables were combined, the AUC value increased above 0.6. These results are consistent with the general concept that an injury is a multifactorial phenomenon and therefore its prediction from a single parameter is not sustainable.

Considering the limitations of the current research, future studies should replicate this design increasing the sample size and over multiple seasons. Moreover, only internal load parameters were quantified, whilst the combination of internal and external load parameters would be an optimal condition.

## PRACTICAL APPLICATIONS

The young players involved in the present study exhibited a different injury rate in relation to maturity status. Particularly, Circa- and Post-PHV players showed the highest injury incidence and injury burden. Coaches and physical trainers should be aware that young players with the same chronological age could exhibit different maturity status, and consequently different physiological responses to training. For this reason, optimising and customising the training loads based on individual players’ needs is crucial to reduce the risk of injuries. Intervention methods suitable for achieving this goal include adequate management of the weekly load, regular monitoring of players’ readiness, and introduction of personalised recovery strategies. Therefore, practitioners should periodically monitor the maturity status of the players, with particular focus on athletes during the PHV.

## CONCLUSIONS

A rapid increase in training load combined with a decrease in TQR represented a dangerous condition for injury and illness risk. More mature players showed a higher perception of effort and lower ability to recover. This may explain the higher injury rate and injury burden found in Circa- and Post-PHV with respect to Pre-PHV players. The internal load markers showed an association with risk of illnesses and non-contact injuries but not predictive ability.

## Funding

The authors received no specific funding for this work.

## Conflicts of interest/Competing interests

The authors have declared that no conflicts/competing interests exist.

## Contributorship

MM, AT and AF were responsible for the conception and design of the study. MM, AT and AF conducted the data analysis and interpretation. The statistical analysis was carried out by MM, AT, GC. The article was written by MM, AT and GC. All authors contributed to the reviewing of the manuscript.
